# Genetic basis of olfactory cognition: extremely high level of DNA sequence polymorphism in promoter regions of the human olfactory receptor genes revealed using the 1000 Genomes Project dataset

**DOI:** 10.3389/fpsyg.2014.00247

**Published:** 2014-03-24

**Authors:** Elena V. Ignatieva, Victor G. Levitsky, Nikolay S. Yudin, Mikhail P. Moshkin, Nikolay A. Kolchanov

**Affiliations:** ^1^Laboratory of Evolutionary Bioinformatics and Theoretical Genetics, Institute of Cytology and Genetics, Siberian Branch, Russian Academy of SciencesNovosibirsk, Russia; ^2^Department of Natural Science, Novosibirsk State UniversityNovosibirsk, Russia; ^3^Laboratory of Molecular-Genetic Systems, Institute of Cytology and Genetics, Siberian Branch, Russian Academy of SciencesNovosibirsk, Russia; ^4^Laboratory of Human Molecular Genetics, Institute of Cytology and Genetics, Siberian Branch, Russian Academy of SciencesNovosibirsk, Russia; ^5^Laboratory of Mammalian Ecological Genetics, Institute of Cytology and Genetics, Siberian Branch, Russian Academy of SciencesNovosibirsk, Russia; ^6^Department of Systems Biology, Institute of Cytology and Genetics, Siberian Branch, Russian Academy of SciencesNovosibirsk, Russia; ^7^National Research centre “Kurchatov Institute”Moscow, Russia

**Keywords:** olfactory cognition, olfactory receptor gene, single nucleotide polymorphism, promoter, 1000 Genomes Project

## Abstract

The molecular mechanism of olfactory cognition is very complicated. Olfactory cognition is initiated by olfactory receptor proteins (odorant receptors), which are activated by olfactory stimuli (ligands). Olfactory receptors are the initial player in the signal transduction cascade producing a nerve impulse, which is transmitted to the brain. The sensitivity to a particular ligand depends on the expression level of multiple proteins involved in the process of olfactory cognition: olfactory receptor proteins, proteins that participate in signal transduction cascade, etc. The expression level of each gene is controlled by its regulatory regions, and especially, by the promoter [a region of DNA about 100–1000 base pairs long located upstream of the transcription start site (TSS)]. We analyzed single nucleotide polymorphisms using human whole-genome data from the 1000 Genomes Project and revealed an extremely high level of single nucleotide polymorphisms in promoter regions of olfactory receptor genes and *HLA* genes. We hypothesized that the high level of polymorphisms in olfactory receptor promoters was responsible for the diversity in regulatory mechanisms controlling the expression levels of olfactory receptor proteins. Such diversity of regulatory mechanisms may cause the great variability of olfactory cognition of numerous environmental olfactory stimuli perceived by human beings (air pollutants, human body odors, odors in culinary etc.). In turn, this variability may provide a wide range of emotional and behavioral reactions related to the vast variety of olfactory stimuli.

## Introduction

Human olfactory perception varies enormously among individuals. People vary both in their general olfactory acuity and in perceiving specific odors. For example, according to a study of 391 adult subjects in New York, general olfactory acuity correlated with age, gender, race, smoking habits, and body type. Factors found to influence olfactory perception included race, age, and gender. Over 100 instances in which the intensity or pleasantness perception of an odor varied significantly among demographic groups were described (Keller et al., 2012). Significant differences in the perception of everyday odors were revealed in a Japanese–German cross-cultural study. A close association of pleasantness ratings and edibility judgments was found, suggesting the particular influence of eating habits on odor perception (Ayabe-Kanamura et al., 1998). Notable differences in perceived odor pleasantness were found in children with autism spectrum disorders: patients with this disorder perceived the smell of cinnamon and pineapple as significantly less pleasant compared to healthy controls, the same was true of cloves (Hrdlicka et al., 2011). Factors influencing human odor perception are extensively studied (Moshkin et al., 2011; Seo et al., 2011, 2013; Greenberg et al., 2013). Recent studies demonstrate that genetic factors may contribute to inter-individual differences in odor perception (Keller et al., 2007; Weiss et al., 2011; Knaapila et al., 2012; Mainland et al., 2014).

The molecular mechanism of olfactory cognition is very complex. In mammals, the cellular and molecular machinery for olfactory transduction is located in olfactory epithelium in the nasal cavity. Odorant transduction is initiated by olfactory (odorant) receptors (ORs), which are located on the membranes of the cilia that are whip-like extensions of olfactory sensory neurons.

Odorants in the mucus bind directly (or are shuttled via odorant-binding proteins) to receptor molecules located in the membranes of the cilia (Supplementary section, Figure S1). The ligand-bound receptor activates the signal transduction cascade, which involves G protein (an olfactory specific subtype, G_olf_), adenylyl cyclase (AC), the cyclic nucleotide-gated (CNG) ion channel and several other proteins (Firestein, 2001; De Palo et al., 2012). Calmodulin (CALM), phosphodiesterase (PDE), β-arrestin2 (ARRB2), some kinases (PKA, GRK3, ORK), and RGS2 protein (regulator of G-protein signaling) participate in feedback mechanisms that olfactory sensory neurons use for adjusting their sensitivity (Boekhoff et al., 1997; Sinnarajah et al., 2001; Mashukova et al., 2006; De Palo et al., 2012). A detailed description of this complex intracellular mechanism is presented in Supplementary section (Part 1).

Mammals have 6–10 million olfactory receptor neurons, which enable organisms to detect and discriminate thousands of odors (Buck and Axel, 1991; Firestein, 2001; Glusman et al., 2001; Olender et al., 2008). There are about 1000 olfactory receptor genes and pseudogenes in the mammalian genome; thus, it is the largest gene family in the entire genome (Firestein, 2001; Menashe et al., 2006). However, in the human genome about 60% of OR genes seem to be pseudogenes (Gilad et al., 2003; Malnic et al., 2004; Hasin et al., 2008; Olender et al., 2012). Their genomic locations show that OR genes are unevenly distributed among 51 different loci on 21 human chromosomes. Sequence comparisons show that the human OR family is composed of 172 subfamilies. Types of odorant structures that can be recognized by some OR subfamilies and OR gene loci were predicted. (Malnic et al., 2004). Analysis of interaction profiles for 93 odorants against 219 murine and 245 human ORs gave rise to a predictive model relating physicochemical odorant properties, OR sequences, and their interactions (Saito et al., 2009). The model was based on 18 physicochemical odorant descriptors and properties of 16 OR amino acid residues. It provided a basis for translating odorants into receptor neuron responses.

Detection of the enormous range of odors requires a combinatorial strategy. Most odor molecules are recognized by more than one receptor (perhaps by dozens), and most receptors recognize several odors, probably related by chemical properties (Firestein, 2001). Each odorant receptor detects distinct sets of odorant molecules. Different odors activate overlapping but non-identical patterns of receptors. The cognition of each odor is based on the detection of signals from different sets of ORs. Two unique structural and functional features of the olfactory system enable an ability of the living organism to discriminate a large number of diverse stimuli. First, each mammalian olfactory sensory neuron expresses only one of ~1000 OR genes (Lewcock and Reed, 2004; Nguyen et al., 2007) In addition, axons from all the cells expressing that particular receptor (no matter where they are found on the epithelial sheet) converge to a single “target” in the olfactory bulb. These targets are glomeruli, spherical conglomerates of neuropils some 0.05–0.1 mm in diameter that consist of the incoming axons of olfactory sensory neurons and the dendrites of the main projection cell in the bulb, the mitral cell (Firestein, 2001).

The sensitivity to a particular ligand depends on the expression level of multiple proteins involved in olfactory cognition: olfactory receptors, proteins that participate in the signal transduction cascade, etc. The content of each protein in the cell is controlled by the expression level of the respective gene.

Transcription is the first step of gene expression at which a particular segment of DNA is copied into RNA by the complex enzyme, RNA polymerase. Transcription is precisely regulated depending on cellular conditions. The transcriptional activity of each gene is regulated by its promoter region which is located upstream of the transcription start site (TSS). Promoters contain specific DNA sequences (transcription factor binding sites), short regions of DNA (10-20 nucleotides) recognized by regulatory proteins (transcription factors). Specific interaction of transcription factors with DNA sequences within promoter region (alone or with other proteins in a complex) facilitates the recruitment of RNA polymerase to specific genes (Merkulova et al., 2013).

Eukaryotic gene regulatory regions may be organized in a complicated manner, so that the regulatory regions of a specific gene may contain binding sites for more than 20 different transcription factors (Kolchanov et al., 2000, 2002, 2008; Vaskin et al., 2011–2012). On the other hand, a great number of different regulatory proteins are involved in transcription regulation. For instance, according to recent data, the human genome encodes about 1500 transcription factors (Zhang et al., 2012; Wingender et al., 2013).

The human olfactory receptor promoters have not been studied sufficiently. Recently, the promoter architecture was characterized in details for 87.5% of the mouse OR genes. (Plessy et al., 2012). It was found that 88.5% of OR promoters were of the sharp type with only a one dominant TSS position (a known feature of tissue-restricted transcripts). Moreover, 21% of OR promoters had a canonical TATA-box (binding site for TATA-binding protein). The binding of the TATA-binding protein (TBP), early B-cell factor 1 (EBF1), and myocyte-specific enhancer factor 2A (MEF2A) to OR promoters was confirmed by chromatin immunoprecipitation. The results of these experiments suggested that transcription factors TBP, EBF1 (OLF1), and MEF2A were involved in the regulation of OR expression.

A single nucleotide polymorphism, or SNP, is a variation at a single position in a DNA sequence among individuals. The 1000 Genomes Project characterizes human genomic variation by using next-generation sequencing strategies. At present, the project reports on genomes of 1092 individuals sampled from 14 populations drawn from Europe, East Asia, sub-Saharan Africa and the Americas. Over 38 million SNPs have been identified by the 1000 Genomes Project, 58.6% of which were previously undescribed (1000 Genomes Project Consortium et al., 2012). According to NCBI's dbSNP build 138 (http://www.ncbi.nlm.nih.gov/SNP/), more than half of the total number of SNPs (59.05%), identified by 1000 Genomes Project, are located in transcribed regions of the human genome, among which 1.07% of the total number are located in coding regions (exons). Of the total number of SNPs, 1.05% are located within the promoter regions of genes. The SNP density in the 500 base pair regions upstream of TSSs is approximately the same as in introns (3.7 SNPs per 1000 bp). It is considerably higher than in coding regions (2.4 SNPs per 1000 bp).

Many SNPs located in the upstream regions of genes are likely to be regulatory. One functional mechanism is that the genetic variants within upstream regions may influence gene transcription by altering the binding affinity of a transcription factor to the DNA (Chorley et al., 2008; Kim et al., 2008; Benson et al., 2011). For example, it was estimated that the G→T substitution (rs1271572) in the *ERβ* promoter prevented transcription factor Yin Yang 1 (YY1) binding and reduced its transcription activity. The TT genotype for rs1271572 was associated with increased risk for breast cancer in Chinese women and with unfavorable prognosis in Chinese breast cancer patients (Chen et al., 2013).

In the other study the T(−13,910) variant upstream the lactase-phlorizin hydrolase gene (*LPH*) associated with lactase persistence was found to bind the octamer transcription factor 1 (Oct-1) tighter than the C(−13,910) variant did. The data suggest that the binding of Oct-1 to the T(−13,910) variant directs elevated lactase promoter activity and this might provide an explanation for the lactase persistence phenotype in the human population (Lewinsky et al., 2005).

Two SNPs (T-1993C and T-1514C) in the promoter of the T box 21 (*TBX21*) gene involved in control of gene expression in T cells have been shown to be associated with systemic lupus erythematosus. Both promoter SNPs effect gene expression by modulating the affinity of a transcription factor binding sites. The affinity of the USF-1 transcription factor (upstream stimulatory factor 1) to the −1514C allele probe was higher than that to the −1514T allele probe. Individuals carrying the −1514C allele were found to have significantly reduced expression of *TBX21* in comparison to those with −1514T allele (Li et al., 2012). In a similar manner, an effect of the T-1993C SNP on the Yin Yang 1 transcription factor-mediated promoter activity was demonstrated (Li et al., 2011).

As discussed above, odor discrimination begins with interaction of volatile organic compounds with different types of low-selective olfactory receptors, inducing different patterns of glomerular activity. Therefore, the patterns of glomerular activity rather than the activities of individual olfactory sensory neurons enable living organisms to recognize odors. Thus, the variability in expression levels of OR genes caused by SNPs located in promoter regions may partly explain the variability of olfactory cognition of different olfactory stimuli and interindividual differences in olfactory perception that are observed in human populations.

The aim of the study was to analyze single nucleotide polymorphisms in promoter regions of human genes controlling olfactory cognition and transduction of olfactory stimuli in olfactory sensory neurons. Using data from the 1000 Genomes Project Consortium we found that 5.5% of human transcripts possessed extremely high SNPs contents in their upstream regions (six and more SNPs per 500-bp region). Functional analysis of this group of transcripts (genes) revealed a large portion of genes involved in olfactory transduction and antigen processing and presentation. Most of genes related to these two biological processes that have six or more SNPs per 500-bp upstream regions were found to belong to the olfactory receptor or HLA gene families. Then comparisons among all genes responsible for olfactory transduction (or antigen processing and presentation, or olfactory receptors only) and genes from the whole genome were done. Analysis of transcript distributions as a function of SNPs contents per 500-bp regions showed that SNP contents for all three functional groups of genes (transcripts) were higher than that for the whole genome set of transcripts. In addition, a similar analysis was performed for longer regions upstream TSSs (1000-bp long) and regions upstream coding region starts (CRSs). An increased genetic variability of upstream regions controlling olfactory transduction and antigen processing and presentation was also observed in these cases.

## Materials and methods

The annotations of genes and SNPs for hg19 assembly of the human genome were extracted from the UCSC Table Browser (https://genome.ucsc.edu/cgi-bin/hgTables, the tracks *hg19 RefSeq genes* and *common SNPs(138)*, respectively; the latter track refers to the release 138 of dbSNP, http://www.ncbi.nlm.nih.gov/SNP). For SNP data, we used additional flags *class single* and *validation by 1000-genomes*. We chose 23,372 transcripts according to the following criteria: (a) only curated transcripts remained in analysis (accession numbers start with NM_, http://www.ncbi.nlm.nih.gov/books/NBK21091/); (b) only data mapped to chromosomes 1–22, X and Y remained in analysis; (c) if at least two transcripts have matching TSSs then only one of them is analyzed. Among selected 23,372 transcripts, 22,290 ones had annotated 5′-untranslated regions (5′UTRs), which means that for 22,290 transcripts positions of TSSs and coding region starts (CRSs) were different. Transcripts were annotated by the length of their 5′UTRs and gene names. We intentionally left in analysis transcripts with matching TSSs and CRSs (see Table 1, line *Whole-genome*). Finally, for each transcript the SNP content was determined as the count of SNPs in the 500 bp long region upstream of the annotated TSS.

**Table 1:** **The description of sequence sets used in analysis and their classification according to number of unique transcripts or genes and the presence/absence of annotated 5′-untranslated regions (5′UTRs)**.

**Dataset of transcripts**		**Number of transcripts/genes**		
**Full name**	**Short name**	**Total**	**5′UTR is annotated**	**5′UTR is not annotated**
Whole-genome		23,372/18,974	22,290/17,961	1082/1013
KEGG pathway *Olfactory transduction*	KEGG_Olf_Tr	414/399	104/92	310/307
KEGG pathway *Antigen processing and presentation*	KEGG_Ant_Pr_Pr	76/70	76/70	0/0
Olfactory receptor genes from HORDE	HORDE_ORs	375/372	62/62	313/310

The DAVID (Database for Annotation, Visualization and Integrated Discovery) web-based Functional Annotation Tool (DAVID tool) was applied (Huang da et al., 2007) to the set of 1258 transcripts, each containing at least six SNPs in the 500-bp region upstream the annotated TSS. The latter dataset will be designated below as *SNP-rich*. The DAVID tool performs functional analysis of large gene lists using information from GO (Gene Ontology) and KEGG (Kyoto Encyclopedia of Genes and Genomes) pathway databases. In GO, genes are annotated using a fixed vocabulary for the description of (a) biological processes in which a gene product is involved, (b) molecular functions which it executes, and (c) cellular compartments in which it is located. The GO vocabulary itself comprises more than 8000 explicitly defined terms and relations between them. The benefits of using the ontological and pathway analyses for functional annotation of group of genes revealed by different criteria have been presented in numerous publications (Smirnova et al., 2009; Jia and Zhao, 2012). The DAVID tool, which was applied for our purpose, allows detection of enriched functionally related gene groups for any specified gene list.

The result was obtained as a *Functional Annotation Chart*, which presents: (a) the list of enriched GO terms and KEGG pathways associated with the gene list; (b) the numbers of genes involved in each GO term or KEGG pathway; (c) fold enrichments for each GO term or KEGG pathway; and (d) the *P*-values for each GO term (or KEGG pathway). A Fold Enrichment is defined by DAVID tool as the ratio of two proportions: the proportion of genes with the GO category (or involved in the KEGG pathway) in a gene list under study, and the proportion of genes associated with the GO category (KEGG pathway) in the human genome. Usually, groups with fold enrichments 1.5 or more are considered to be interesting (Huang da et al., 2009). The enriched GO terms from the *biological processes* vocabulary were considered in our study. The significance of GO terms (and biological pathways) is estimated by DAVID tool on base of the number of genes from the list under study and the number of genes expected by chance. The significance of GO terms (or biological pathways) was estimated through the EASE score, a modified Fisher exact *p*-value (a built-in function of DAVID tool). The standard significance level *p* < 0.05 was applied. The count threshold value was 2 and the EASE threshold value was 0.1.

Another approach was based on the analysis of distribution of the SNP content in 500-bp long upstream regions of human genes from KEGG pathways (http://www.genome.jp/kegg/pathway.html). KEGG provides a large collection of manually derived schemes of metabolic and signaling pathways, as well as of a variety of related diseases and other processes. Namely, the pathways *Olfactory transduction* (Pathway_ID—hsa04740) and *Antigen processing and presentation* (Pathway_ID—hsa04612) were considered. In addition, the group of genes encoding ORs was considered. To estimate the promoter variability for genes encoding ORs, genes encoding ORs were extracted from HORDE (The Human Olfactory Data Explorer, http://genome.weizmann.ac.il/horde/) (Olender et al., 2013).

Final lists of transcripts for three groups were compiled according to criteria a, b, c, described above in this section for the whole-genome set of transcripts. The corrected numbers of transcripts/genes for all groups are given in Table 1.

The distributions of SNP contents for 500-bp long upstream regions for any *k*^th^ group (*k* = 1, 2, 3) of transcripts were compared to that for the whole-genome dataset. The statistical significance of differences was estimated by Welch's *t*-test for angular (arcsine square root) transformed proportions (Sokal and Rohlf, 1995). The first proportion *p*_1,*n(k)*_ was computed as the ratio of the number of transcripts having at least *N* SNPs in upstream regions to the total number of transcripts in *k*^th^ group. The second proportion *p*_2,*n*_ was calculated similarly for the whole genome dataset. For the range of thresholds *N* (from 1 to 20) the angular transformation *y*(*p_i,n_*) was computed to apply the *t-*test as follows: $y_{i}=2\arcsin\left(\sqrt{p_{i}}\right)$, where *i* = 1, 2. Additionally, in order to take into account missed annotations of 5'UTRs in some transcripts (Table 1), similarly to the aforementioned pipeline for analysis of 500-bp regions upstream TSSs we performed the corresponding analysis for: (a) 1000-bp regions upstream TSSs, (b) 500-bp regions upstream CRSs; in the next cases only transcripts with distinct annotated TSSs and CRSs were remained in analysis, (c) 500-bp regions upstream TSSs, and (d) 500/1000-bp regions upstream CRSs.

## Results

### Human promoter variability for the whole genome dataset

**Figure 1:**
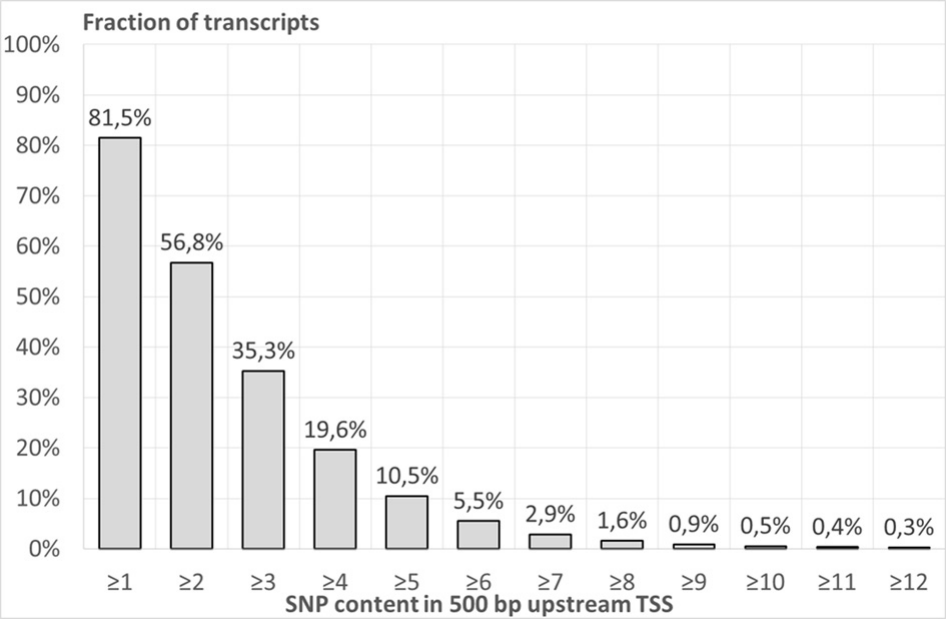
**The fraction of human transcripts from the whole genome dataset (Table 1), possessing at least certain number of SNPs in 500-bp long regions upstream annotated transcription start sites**. X axis—SNP content in 500 bp upstream TSS. Y axis—fraction of the whole genome dataset of transcripts.

Figure 1 shows the fractions of human transcripts (from the whole genome dataset of 23,372 transcripts, see Materials and Methods), possessing at least certain numbers of SNPs (SNP content) in 500-bp long regions upstream annotated TSSs. This number of SNPs is designated as the threshold for the SNP content in upstream region and is marked on the X-axis. The majority of transcripts have low or intermediate SNP contents in their 500-bp regions upstream annotated TSSs. For example, at least one SNP was found in the upstream regions of 81.5% of transcripts. This means that the other transcripts of the whole genome dataset (18.5%) do not contain SNP in their 500-bp long upstream regions. At least two SNPs were observed in the upstream regions of 15,149 (56.8%) transcripts. However, at least six SNPs were found in 1,258 (5.5%) transcripts. As it was mentioned in Materials and Methods, this set of transcripts was designated as *SNP-rich*. The highest SNP content (53 SNPs) was found in the *HLA-DQA1* gene. Table S1 presents the list of all transcripts from the whole-genome dataset with the respective SNP contents in their 500-bp upstream regions.

### Biological processes and pathways overrepresented among genes whose transcripts were found in the SNP-rich dataset

GO terms and biological pathways, which were overrepresented among genes whose transcripts were found to have at least six SNPs in their 500-bp long upstream regions (*SNP-rich dataset*) were selected by applying DAVID tool. As described in Materials and Methods DAVID annotation implies the classification of gene product function by relevant GO terms or KEGG pathway. Hence, in such a way, the gene sets could be functionally annotated and enriched GO terms and biologically relevant pathways could be identified. Inspection of GO categories overrepresented in the *SNP-rich dataset* revealed two biological processes among the top ones: *sensory perception of smell* and *antigen processing and presentation*. In both cases, fold enrichment exceeded 1.5, and *P*-Values were less than 0.01 (Table 2).

**Table 2:** **Biological processes overrepresented (*p* < 0.05) in the *SNP-rich dataset*, which includes transcripts with at least six SNPs in 500-bp upstream regions**.

**Biological process (GO category)**	**Number of genes from the *SNP-rich dataset* annotated by the category**	**Fold enrichment**	***p*-Value**	**Genes**
GO:0007608~sensory perception of smell	48	1.7	1.98E-04	*OR10A5, OR52H1, OR51B2, OR1L8, OR1A2, OR5D16, OR52J3, OR51F1, OR1N2, OR8S1, OR1L3, OR11G2, OR13C5, OR4C16, OR10G9, OR8I2, OR7C2, OR51L1, OR2C3, OR8G1, OR14C36, OR2AG1, OR2B11, OR8G5, OR11L1, OR4M2, OR6C74, OR9G1, OR6C75, OR6T1, OR2W3, OR51G2, OR10Z1, OR12D2, OR5H14, OR10AD1, OR52E6, OR11H6, OR4A15, OR5H15, OR10Q1, OR4C45, OR5H6, OR7D4, OR6K6, OBP2A, PDE1C, GNAL*
GO:0019882~antigen processing and presentation	18	3.32	3.95 E-05	*HLA-DQB1, HLA-DRB1, HLA-A, HLA-C, HLA-B, HLA-DQA2, HLA-G, HLA-DQA1, HLA-F, HLA-DRB5, HLA-DPA1, HLA-DRA, MICB, MICA, LOC554223, TAP2, ULBP2, CTSE*

According to the DAVID report, the group of genes annotated by the GO term *sensory perception of smell* includes 45 genes encoding odorant receptors and three other genes: *OBP2A* (Odorant-binding protein 2a); *GNAL* (guanine nucleotide binding protein (G protein), alpha activating activity polypeptide, olfactory type); and *PDE1C* (phosphodiesterase 1C, calmodulin-dependent 70kDa). The upstream region of *OR9G1* (olfactory receptor, family 9, subfamily G, member 1 gene) was extremely variable, containing 15 SNPs per 500 bp.

Eighteen genes from *SNP-rich dataset* were annotated by the GO category antigen processing and presentation. Among them, 12 genes (*HLA-DQB1, HLA-DRB1, HLA-A, HLA-C, HLA-B, HLA-DQA2, HLA-G, HLA-DQA1, HLA-F, HLA-DRB5, HLA-DPA1, HLA-DRA*) belonged to the HLA gene family, and six genes (*MICB, MICA, LOC554223, TAP2, ULBP2, CTSE*) belonged to other families. This group contained two genes (*HLA-DQA1, HLA-B*) that had the highest promoter SNP contents (53 and 29, respectively) among all genes from the whole-genome dataset.

Inspection of KEGG pathways whose genes were overrepresented in the *SNP-rich dataset* identified two top pathways: *Olfactory transduction* (Pathway_ID—hsa04740) and *Antigen processing and presentation* (Pathway_ID—hsa04612). Since in both cases the fold enrichment exceeds 1.5 and *P*-Value is below 0.01 (Table 3), we conclude that genes from these two KEGG pathways are significantly overrepresented in the *SNP-rich dataset*. A substantial fraction of genes (96%, or 43 of 45) found to be involved in the olfactory transduction pathway were recognized as olfactory receptor genes. A half of genes (12 of 22) involved in antigen processing and presentation pathway belonged to the family of genes called the human leukocyte antigen (*HLA*) complex.

**Table 3:** **Biological pathways overrepresented (*p* < 0.05) in the *SNP-rich dataset*, which includes transcripts with at least six SNPs in their 500-bp upstream regions**.

**KEGG pathway**	**Number of genes from the *SNP-rich dataset* belonging to the pathway**	**Fold enrichment**	***p*-Value**	**Genes**
*Olfactory transduction* (Pathway_ID—hsa04740)	45	1.68	6.37E-04	*OR10A5, OR52H1, OR51B2, OR1L8, OR1A2, OR5D16, OR52J3, OR51F1, OR1N2, OR8S1, OR1L3, OR11G2, OR13C5, OR4C16, OR10G9, OR8I2, OR51L1, OR7C2, OR2C3, OR8G1, OR14C36, OR2AG1, OR2B11, OR8G5, OR11L1, OR4M2, OR6C74, OR9G1, OR6C75, OR6T1, OR2W3, OR51G2, OR10Z1, OR12D2, OR10AD1, OR52E6, OR11H6, OR4A15, OR4C45, OR10Q1, OR5H6, OR7D4, OR6K6, GNAL, PDE1C*
*Antigen processing and presentation* (Pathway_ID—hsa04612)	24	4.0	2.11E-08	*HLA-DQB1, HLA-DRB1, HLA-A, HLA-C, HLA-B, HLA-DQA2, HLA-G, HLA-DQA1, HLA-F, HLA-DRB5, HLA-DPA1, HLA-DRA, HSP90AB1, IFNA21, KLRC3, IFNA10, KIR2DS4, HSPA2, TAP2, TAP1, HSPA6, IFNA16, IFNA17, CD4*

### Promoter variability for genes controlling olfactory transduction, antigen processing, and presentation and genes encoding olfactory receptors

Our second analysis was undertaken to compare promoter variability for genes controlling olfactory transduction or antigen processing and presentation to that for the whole-genome dataset. The lists of genes, belonging to these two pathways were extracted from the KEGG database. These lists are denoted below as *KEGG_Olf_Tr* and *KEGG_Ant_Pr_Pr*, respectively (Table 1).

Since olfactory receptor genes comprise a large fraction of genes detected in the *SNP-rich dataset* by GO and pathway analysis, it was interesting to analyze promoter variability for this genes. For this purpose, the gene list *HORDE_ORs* was compiled using data from HORDE (Table 1).

**Figure 2:**
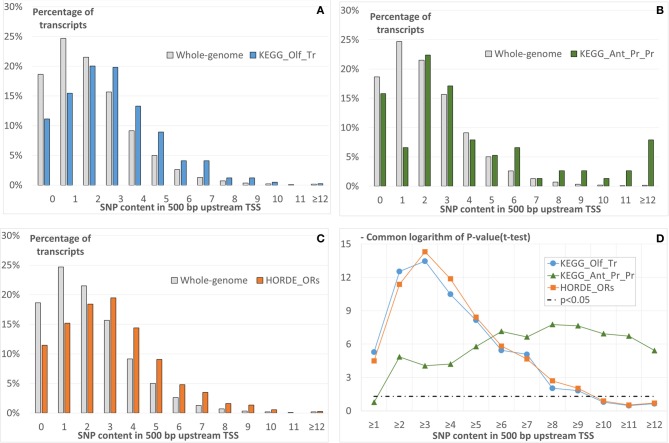
**The comparison of SNP content distributions in upstream regions of three groups of human transcripts with that for whole-genome dataset (Table 1)**. First 500-bp upstream annotated TSSs are analyzed. The groups are derived from KEGG pathways **(A)** *Olfactory transduction*, **(B)** *Antigen processing and presentation*, and **(C)** from HORDE. In panels from **(A** to **C)** the X axis denotes the SNP content; the Y axis, the count of transcripts with specific SNPs in the upstream regions. Panel **(D)** presents the significances of the *t*-test (Y axis), which compare the above-described SNP contents in three groups with that for the whole genome dataset as a function of the SNP content (X axis). The *t-*test was applied as described in Materials and Methods.

The comparison of distribution of the SNP content in 500-bp long regions upstream annotated TSSs in either group of transcripts and that for the whole-genome dataset shows that transcripts of all groups tend to have higher SNP contents (Figures 2A–C). To confirm this conclusion, we applied the *t-test* for angular transformed proportions (see Materials and Methods). This test was applied for the range of thresholds of SNP content (Figure 2D). We concluded that for any threshold of SNP content from one to nine the significant enrichment of transcripts with SNPs was observed for all the three gene groups.

To ensure that missed annotation of 5′UTRs for transcripts of whole genome dataset, and especially for transcripts of *HORDE_ORs* and *KEGG_Olf_Tr* groups (Table 1) cannot substantially influence our conclusions, we performed additional analyses.

Below we will use the designations (−1 kb; TSS), (−500; TSS), (−1 kb; CRS), (−500; CRS) for 1000-bp or 500-bp regions upstream TSSs or CRSs. The prefixes 5′UTR ≥ 0 or 5′UTR > 0 mean that a 5′UTR may have any length, or only a positive value is allowed (i.e., the TSS and CRS positions are different). In these terms, a pipeline analysis in the case 5′UTR ≥ 0_(−500; TSS) is presented above in this section (Figure 2).

The results, similar to those depicted in Figure 2D, i.e., dependencies of the significance of the *t-*test on the threshold of SNP contents for the different combinations of upstream region lengths and locations and on 5′UTR′s annotation availability are presented in Figure S2. We came to the following conclusions:

- 5′UTR ≥ 0_(−1 kb; TSS) case (Figure S2A). We observed the pattern similar to that in the 5′UTR > 0_(−500; TSS) case; however, the group of smallest size (*KEGG_Ant_Pr_Pr*) revealed very moderate significance of the *t-*test (*p* < 0.02 for at least eight SNPs).
- 5′UTR > 0_(−500; TSS) case. All groups show less significant results than in the 5′UTR ≥ 0_(−500; TSS) case described above (Figure 2D); nevertheless the significance *p* < 0.05 is observed for SNP content thresholds from two to six.
- 5′UTR ≥ 0_(−500; CRS) case. The overall results are similar to those described previously for the case 5′UTR ≥ 0_(−500; TSS).
- The 5′UTR > 0_(−500; CRS) and 5′UTR > 0_(−1 kb; CRS) cases show that only for the latter case an SNPs enrichment is observed for all three test groups (Figures S2D,E).

The 5′UTR ≥0 (−1 kb; TSS) case proves that the enrichment of upstream regions with SNPs hardly can strongly depend on region length. In the 5′UTR > 0_(−500; TSS) case, we are sure that the enrichment is related to the promoter region of a gene. The 5′UTR ≥ 0_(−500; CRS) case allows us to suppose that, as in case of promoter regions, 5′UTRs also have an enrichment of SNPs for transcripts of all three groups under study. However, the cases 5′UTR > 0_(−500; CRS) and 5′UTR > 0_(−1 kb; CRS) argue for the major impact of promoter region in the enrichment of SNPs in the upstream regions of transcripts classified into three groups.

## Discussion

### Increased genetic variability in the promoter regions of genes controlling sensory perception of smell and antigen processing and presentation

Our study revealed a broad variability of SNP contents in promoters of genes from the whole-genome dataset. Almost a one-fifth (18.5%) of the total number of promoters had no SNPs at all (Figure 1). However, a very interesting set of promoters characterized by high SNP contents (six or more SNPs) was found. Among the genes with high SNP content in promoters, three groups were overrepresented according to the DAVID tool (Huang da et al., 2007): (1) genes controlling the sensory perception of smell; (2) a specific subset of promoters of sensory perception genes encoding olfactory receptors (ORs), and (3) genes involved in antigen processing and presentation (Tables 2, 3). We compared the contents of SNPs in the upstream regions of genes of the aforementioned groups with that for the whole genome dataset by Welch's *t-*test for angular transformed proportions. It was shown that promoters of all three groups were characterized by increased genetic variability in comparison to that for the whole genome dataset. The detailed analysis showed that regions located both upstream and immediately downstream the transcription start, participated in SNPs enrichment (Figure S2). The clarification of this issue is still hampered by the scarce annotation of TSSs in genome. Nevertheless, the importance of 5′UTRs for transcription regulation is still underestimated (Omelina et al., 2011).

### Parallelism between olfactory cognition and functions of the immune system (ability to distinguish between self and non-self)

The whole-genome analysis of the SNP content in promoter DNA revealed two interesting groups of genes with the highest genetic variability: genes controlling sensory perception of smell and genes responsible for antigen processing and presentation. Actually, the biological functions of these two systems are similar. As far back as 1975, parallelism and even adaptive molecular convergence between olfactory cognition, on the one hand, and the heart of the immune system, its ability to distinguish between self and non-self, was found (Thomas, 1975). Both systems are targeted on the reception of extremely variable chemical compounds in the environment of living organisms and immune recognition of parasitic and commensal microbiotas, which evolve very rapidly. Therefore, it is not surprising that genes of both these systems have the highest promoter SNP contents among all genes in the human genome. Such extremely high variability may cause diversity in the expression levels of olfactory receptors and genes of the immune system as well. Recently, it has been suggested that OR diversity is maintained to an extent by balancing selection, similar to that acting upon the major histocompatibility complex alleles at the population level (Olender et al., 2012). Our results suggest that regulatory regions of OR genes and genes responsible for antigen processing and presentation may also be under such selection.

### Genetic diversity in coding regions of OR genes and variations in odor perception

The ability to detect many odors varies among individuals; however, the contribution of genotype to this variation has been assessed for relatively few compounds. Several recent studies demonstrate that the genetic variation in the coding regions of human OR genes contributes to the variation in odor perception among individuals.

The human odorant receptor, *OR7D4*, is selectively activated *in vitro* by androstenone and the related odorous steroid androstadienone (androsta-4,16-dien-3-one), and it does not respond to a panel of other 64 odors and two solvents. Genotypic variation in *OR7D4* accounts for a significant proportion of the valence (pleasantness or unpleasantness) and intensity variance in perception of these steroidal odors. A common variant of this receptor contains two non-synonymous SNPs, resulting in two amino acid substitutions (R88W, T133M; hence ‘RT’) that severely impair its function *in vitro*. Human subjects with RT/WM or WM/WM genotypes were less sensitive to androstenone and androstadienone, and they found both odors less unpleasant than the RT/RT group did (Keller et al., 2007). Since androstenone is naturally present in meat derived from male pigs, the study evaluating the effect of two non-synonymous SNPs in *OR7D4* gene on food preferences was carried out. When pork containing varying levels of androstenone was cooked and tested by sniffing and tasting, subjects with two copies of the RT variant tended to rate the androstenone-containing meat as less favorable than subjects carrying the WM variant (Lunde et al., 2012). It was also found that the genetic variation in *OR7D4* (variant rs8109935) may influence odor perception (pleasantness/unpleasantness) between heterosexual partners (Sookoian et al., 2011).

The genetic basis of odorant-specific variations in human olfactory thresholds, and, in particular, of enhanced odorant sensitivity (hyperosmia) was explored. The association between olfactory detection threshold phenotypes for four odorants and segregating pseudogene genotypes of 43 ORs was examined (Menashe et al., 2007). A strong association signal was observed between the SNP variants in *OR11H7P* and sensitivity to the odorant isovaleric acid. This association was largely due to the low frequency of homozygous pseudogenized genotype in individuals with specific hyperosmia to this odorant, implying a possible functional role of *OR11H7P* in isovaleric acid detection.

Resting on the fact that smoking behavior has been associated in two independent European cohorts with the most common Caucasian human leukocyte antigen (HLA) haplotype (A1-B8-DR3), a study linking smoking to a distinct OR allele was carried out (Santos et al., 2008). The non-synonymous SNP within the *OR12D3* gene (rs3749971) was found to be associated with the HLA haplotype-dependent differential recognition of cigarette smoke components for the Hungarian cohort. This polymorphism leads to a Thr → Ile substitution that affects a putative ligand-binding region of the OR12D3 protein.

A genetic basis for the ability to detect the flavor compound cis-3-hexen-1-ol was determined recently (McRae et al., 2012). This compound is typically described as “green grassy” or the smell of “cut grass.” One SNP variant (rs28757581), found in the coding region of the *OR2J3* gene, was strongly associated with cis-3-hexen-1-ol detection threshold concentrations. This polymorphism encodes a T113A substitution in OR2J3 protein. The *OR2J3* gene contained five predicted haplotypes in the 52 individuals from New Zealand. The majority of the individuals studied were Caucasians (73.6%), and other subjects were Indians (13.2%), Asians (11.3%), and Maoris (1.9%). All five haplotypes were tested *in vitro*. It was shown that two amino acid substitutions, T113A and R226Q, impaired the ability of OR2J3 to respond to *cis*-3-hexen-1-ol, and the presence of both effectively abolished the response to the compound. The haplotype of *OR2J3* containing both T113A and R226Q was responsible for 26.4% of the variation in cis-3-hexen-1-ol detection in the cohort under consideration.

### The biological significance of SNPs located in upstream regions of genes involved in olfactory transduction

Evidence for biological significant variation found in the upstream region of the olfactory receptor 2M7 (*OR2M7*) gene was obtained from two unrelated studies. Thirty-eight adult men and women from Philadelphia (Caucasian; African-American; Asian etc.) participated in the first study (Pelchat et al., 2011). One SNP within a cluster of fifty olfactory receptor genes was found to be associated with the inability to smell the asparagus odor, which is detected in urine of people who have recently eaten asparagus. The urine of these people has a sulfurous odor, which is distinct and similar to cooked cabbage. Asparagusic acid (1,2-dithiolane-4-carboxylic acid) is found in asparagus, and it may be the precursor to some of the sulfur metabolites found in asparagus urine. The most common odorant detected in asparagus urine is methanethiol. The inability to smell the asparagus odor in urine was associated with the variant rs4481887 located upstream the *OR2M7* gene. The A allele was associated with greater ability to detect the asparagus odorant than G. There were racial differences in rs4481887 allele frequency, with Caucasian subjects having a minor allele frequency of 0.35, whereas there was no observed genetic variation in subjects of African descent (all genotypes were GG) (Pelchat et al., 2011). The same allele was associated with the ability to smell the asparagus odor in the second study, which reported results for individuals having European ancestry (Eriksson et al., 2010). Since this SNP is located approximately 9 kb upstream of the *OR2M7* translation initiation codon, these two studies provide the first piece of evidence for significant biological variation found in the upstream region of an olfactory receptor gene. It is conceivable that the nucleotide substitution in this position changes the affinity of some transcription factor to the DNA region containing the SNP, affecting the *OR2M7* gene expression level.

The obvious demonstration that the nucleotide substitution in promoter region of gene from olfactory transduction pathway can alter the binding of a transcription factor and thus result in impaired gene transcription was obtained for the *ADRBK2* gene. *ADRBK2* encodes G-protein-coupled receptor kinase 3R (GRK3) which participates in termination of olfactory signaling, phosphorylating activated olfactory receptors and thus transforming them to the desensitized state (Boekhoff et al., 1997). It was demonstrated that the rare variant of SNP G-384A (rs41261045) disrupts Sp1 transcription factor binding to DNA *in vitro*, and increases *ADRBK2* promoter-driven expression in cell transfection models (Zhou et al., 2008). The rare variant of SNP G-384A was reported to be associated with bipolar disorder in two independent samples (Barrett et al., 2003). However, in this case the possible effect of the G→A nucleotide substitution on olfactory cognition has not been studied.

Two SNPs in the upstream region of *OR51B4* gene were found among genetic modifiers of Hb E/b0 thalassemia identified by a two-stage genome-wide association study (Sherva et al., 2010). Both SNPs were significantly associated with disease severity. One SNP (rs10837774) was less than 500 bp upstream from the start of *OR51B4* transcript. The other (rs3886223) located ~20 kb upstream from *OR51B4* was the most closely associated SNP in this group, with the common allele contributing to increased risk of severe disease in an additive fashion.

Thus, only few studies describe the effects of polymorphisms found in upstream regions of olfactory receptors genes. Nevertheless, investigations of SNPs in the upstream regions of the *OR2M7, OR51B4*, and *ADRBK2* genes involved in olfactory transduction (Zhou et al., 2008; Eriksson et al., 2010; Pelchat et al., 2011) as well as SNPs in the upstream regions of *ERβ, LPH*, *TBX21* and many other genes controlling a variety of cellular processes (Lewinsky et al., 2005; Li et al., 2011, 2012; Chen et al., 2013) show that such SNPs may have a great impact on phenotypic traits.

### The extremely high genetic diversity of human olfactory receptor genes estimated from the 1000 genomes project dataset

Olfactory receptor genes are the largest gene family in the human genome comprising ~400 genes and ~600 pseudogenes (Firestein, 2001; Hasin et al., 2008; Olender et al., 2012). Therefore, ORs may be a special challenge for high-throughput sequencing and genotyping due to the high level of homology observed in their coding regions. Nevertheless, we believe, that the high genetic diversity of upstream regions of OR genes observed in our study could not be explained, entirely or partially, by incorrect assemblage of the olfactory genome.

First, in both phase 1 and pilot stages of the 1000 Genomes project the special filter *depth threshold* was applied to remove miscalling of SNPs based on the mapping of paralogous sequences (1000 Genomes Project Consortium et al., 2010). The filters on coverage and fraction of reads with low mapping quality lead to the exclusion of a substantial fraction of sites in the genome. More details are presented in the Supplementary section (Part 3). We are sure that if the upstream regions of olfactory receptor genes had any assembly problems their SNPs would certainly be excluded from the final SNP set.

Second, an unusually high genetic diversity of genes of the olfactory transduction pathway was described in the 1000 Genomes Project report (1000 Genomes Project Consortium et al., 2012). According to table S13 presented in the Supplementary Information to this report, genes belonging to the KEGG pathway *Olfactory transduction* (of which 92% belong to the odorant receptor family) had the highest SNP content in coding regions (16.9 SNPs per 1000 bp) among examined KEGG pathways. As presented in Figure S11b in the Supplementary Information to 1000 Genomes Project report, the genes from the olfactory transduction pathway had an excessive number of rare non-synonymous SNPs and a high level of conservation in the American ancestry-based group.

Third, an unusually high genetic diversity was found previously in coding regions of human olfactory receptor genes. On average, two individuals have functional differences at over 30% of their odorant receptor alleles (Mainland et al., 2014). The degree of genomic variation for coding regions of OR genes was one SNP per 66 bases, 2.5 times larger than in coding exons of the control genes (Olender et al., 2012). In that study, a comprehensive catalog of genetic variability in the human olfactory receptor genes was compiled. A major resource for this work was the 1000 Genomes Projects whole genome sequence data, and to a lesser extent, dbSNP. The authors performed experimental validation of non-functional SNPs using a custom SNP array (Illumina GoldenGate Genotyping Assay). The final design included 285 non-functional OR variations, of which 268 were successfully genotyped in a cohort of 468 individuals of two ethnicities (validation rate 94%). The majority (65%) of the unsupported variations were mined from dbSNP (Olender et al., 2012). We believe that this high validation rate (94%) revealed for non-functional SNPs in coding regions of OR genes by Olender et al. (2012) confirms the validity of the 1000 Genomes Projects data for all olfactory receptor loci in whole.

## Final conclusions

The majority of investigations of OR genes demonstrate that genetic variability in coding regions of OR genes may be associated with differences in olfactory cognition and odor perception, confirming the idea of functional importance of coding SNPs. The impact of SNPs, located in the 5′regions of OR genes on gene function and phenotype is still defined very poorly. However, the examples considered above demonstrate that (a) some polymorphic alleles in upstream regions of genes involved in olfactory cognition may be associated with variations in odor perception; (b) genetic variation in the promoter region may considerably impair transcriptional regulation of a particular gene, changing morphological, behavioral, physical, and/or biochemical traits of an organism. We suggest that the extremely high SNP content in the promoters of OR genes revealed in our study causes variations in gene expression. In turn, the elevated variability in ORs expression may partly explain individual differences in odor perception.

The extremely high level of the SNP content in promoters of olfactory receptor genes revealed in our study raises the question about the functional significance of such SNPs for olfactory cognition as well as about their association with human diseases. The genome-wide view on human olfaction with the emphasis on regulatory SNPs may provide understanding of some aspects of personalized odor coding. Theoretical analysis of the potential functional role of nucleotide substitutions found in upstream regions of genes may outline possible molecular mechanisms of SNP effects at the gene expression level. These two approaches combined with subsequent experimental verification of theoretical assumptions and hypotheses may be helpful for understanding the molecular mechanism linking olfactory cognition with individual emotional and behavioral reactions to a broad variety of olfactory stimuli: air pollutants, human body odors (including body odors affected by anxiety or bacteria), odors in culinary etc.

## Author contributions

Elena V. Ignatieva, Mikhail P. Moshkin and Nikolay A. Kolchanov designed the study. Elena V. Ignatieva, Victor G. Levitsky performed the study. Elena V. Ignatieva, Victor G. Levitsky, Nikolay S. Yudin were involved in data analysis. Elena V. Ignatieva drafted the manuscript. Victor G. Levitsky, Nikolay S. Yudin, Mikhail P. Moshkin and Nikolay A. Kolchanov corrected the manuscript. All authors read and approved the final manuscript.

### Conflict of interest statement

The authors declare that the research was conducted in the absence of any commercial or financial relationships that could be construed as a potential conflict of interest.

## Abbreviations

5′UTRs: 5′-untranslated regions
bp: base pairs
CRS: coding region start
Kb: kilobase (1000 base pairs of DNA)
OR: olfactory receptor
SNP: single nucleotide polymorphism
TSS: transcription start site
Amino acids: A: Alanine
Ile: Isoleucine
Q: Glutamine
R: Arginine
T: Threonine
W: Tryptophan
M: Methionine
Thr: Threonine
Nucleotides: A: Adenine
C: Cytosine
G: Guanine
T: Thymine.
