# Supplementary material for: Genetic basis of olfactory cognition: extremely high level of DNA sequence polymorphism in promoter regions of the human olfactory receptor genes revealed using the 1000 Genomes Project dataset
Source: Front Psychol. 2014 Mar 24;5:247. doi: 10.3389/fpsyg.2014.00247 (PMC3970011; doi:10.3389/fpsyg.2014.00247)
Supplement: Supplementary file 2 [file DataSheet2.DOCX]

**Supplementary section**

**Part 1. The signal transduction cascade activated by ligand-bound olfactory receptor**

Odorants in the mucus bind directly (or are shuttled via odorant-binding proteins) to receptor molecules located in the membranes of the cilia (Figure S1). The ligand-bound receptor activates G protein (an olfactory specific subtype, Golf, comprising α, β, γ subunits), which, in turn, activates adenylyl cyclase (AC). This enzyme converts the abundant intracellular molecule ATP into cyclic AMP, a molecule that plays numerous signaling roles in cells. In the case of olfactory sensory neurons, cAMP binds to the intracellular face of a cyclic nucleotide-gated (CNG) ion channel, enabling it to conduct cations such as Na^+^ and Ca^2+^. The elevated influx of Na^+^ and Ca^2+^ into the cilia depolarizes them. If a sufficient number of channels are open for long enough, shifting the membrane potential to positive by about 20 mV, the cell reaches a threshold and generates an action potential. This pathway is supplemented by an additional amplification mechanism in olfactory sensory neurons, unique in some respects. The calcium ions entering through the CNG channel can activate another ion channel that is permeable to the negatively charged chloride ion. Chloride channels provide most of the depolarization of the olfactory receptor potential. This depolarization is conducted passively from the cilia to the axon hillock region of the olfactory sensory neuron, where action potentials are generated and transmitted to the olfactory bulb (Firestein 2001).

| 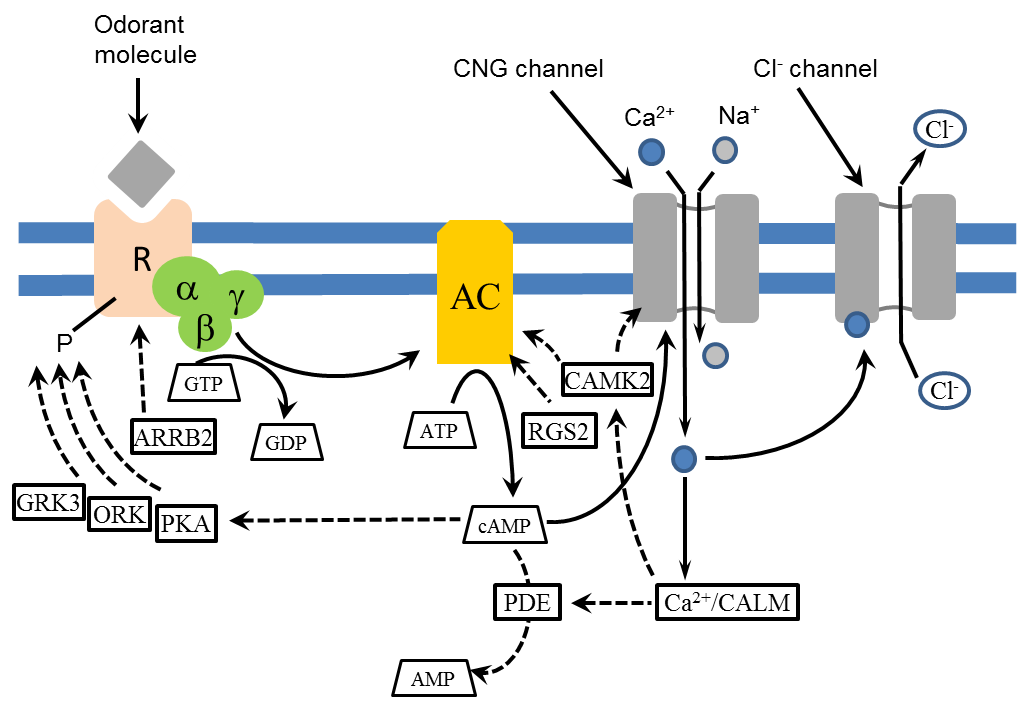 |
| --- |
| Figure S1. The principal scheme of olfactory transduction in an olfactory sensory neuron. Within the compact cilia of the olfactory sensory neuron, a cascade of enzymatic activities transduces the binding of an odorant molecule to a receptor into an electrical signal that can be transmitted to the brain. AC, adenylyl cyclase; CNG channel, cyclic nucleotide-gated channel; PDE, phosphodiesterase; PKA, protein kinase A; ORK, olfactory receptor kinase; GRK3, G-protein-coupled receptor kinase 3R; olfactory receptor; RGS2, regulator of G proteins (but here acts on the AC); CALM, calmodulin; CAMK2, calcium/calmodulin-dependent protein kinase II, α, β, γ, - Golf protein subunits. Trapezoids designate organic phosphates: GTP, guanosine triphosphate; GDP, guanosine diphosphate; ATP, adenosine triphosphate; cAMP, cyclic adenosine monophosphate; AMP, adenosine monophosphate. White rectangles designate inhibitory proteins. Black solid arrows indicate stimulatory pathways; dotted arrows indicate inhibitory pathways (feedback). Modified from (Firestein 2001; De Palo et al., 2012). |

There are several mechanisms that olfactory sensory neurons use for adjusting their sensitivity. Ca^2+^ entry not only amplifies the response by gating Cl^-^ current but also, in combination with Ca-binding proteins, induces feedback mechanisms: (a) Ca-calmodulin (Ca^2+^/CALM) increases the phosphodiesterase (PDE) hydrolysis of cAMP; (b) Ca/CALM activates Ca-calmodulin -dependent protein kinase II (CAMK2), which inhibits AC, and (c) The CALM and/or other Ca-binding proteins are natively bound to CNG channels. Moreover, (a) stimulated ORs stably interact with β-arrestin2 (ARRB2) and undergo clathrin-dependent endocytosis, resulting in their accumulation in endocytic vesicles for extended periods of time (Mashukova et al., 2006); (b) some kinases (PKA, ORK, GRK3) phosphorylate activated receptors sending them into a desensitized state (Boekhoff et al., 1997, De Palo et al., 2012); and (c) RGS2 protein (regulator of G-protein signaling) acts on adenylyl cyclase to decrease its activity (Sinnarajah et al. 2001).

**References**

1. Boekhoff, I., Touhara, K., Danner, S., Inglese, J., Lohs,e M.J., Breer, H., Lefkowitz, R.J. (1997) Phosducin, potential role in modulation of olfactory signaling. *J Biol Chem.* 272, 7, 4606-12.
2. De Palo, G., Boccaccio, A., Miri, A., Menini, A., Altafini, C. (2012) A dynamical feedback model for adaptation in the olfactory transduction pathway. Biophys J. 102, 12, 2677-86.
3. Firestein, S. How the olfactory system makes sense of scents. (2001) *Nature.* 413, 6852, 211-8.
4. Mashukova, A., Spehr, M., Hatt, H., Neuhaus, E.M. (2006) Beta-arrestin2-mediated internalization of mammalian odorant receptors. *J. Neurosci.* 26, 39, 9902-12.
5. Sinnarajah, S., Dessauer, C.W., Srikumar, D., Chen, J., Yuen, J., Yilma, S., Dennis, J.C., Morrison, E.E., Vodyanoy, V., Kehrl, J.H. (2001) RGS2 regulates signal transduction in olfactory neurons by attenuating activation of adenylyl cyclase III. *Nature.* 409, 1051–1055.

**Part 2. Promoter variability for genes controlling olfactory transduction, antigen processing and presentation and genes encoding olfactory receptors: regions located both upstream and immediately downstream the transcription start participate in SNP enrichment**

| 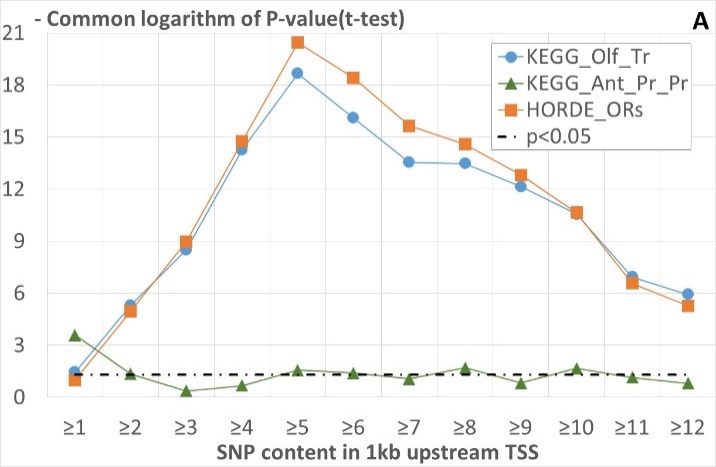 | 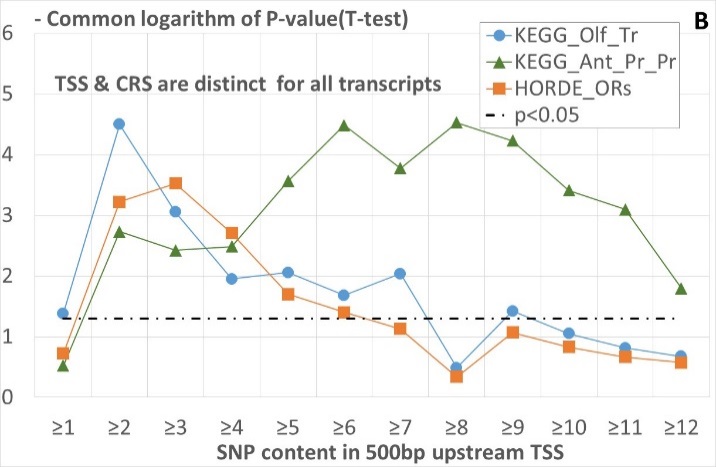 |
| --- | --- |
| 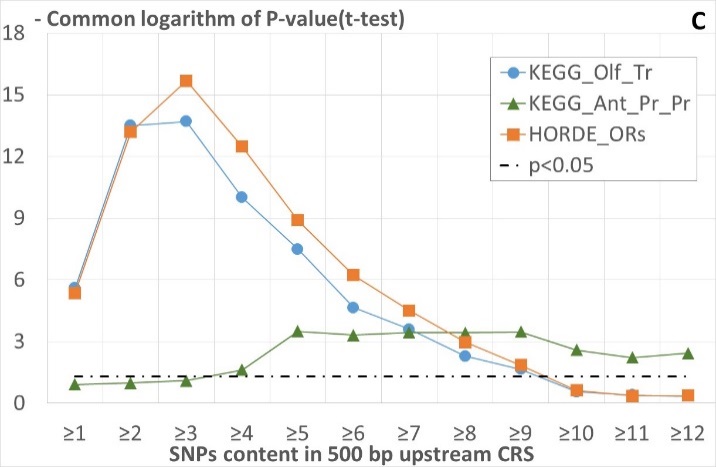 | 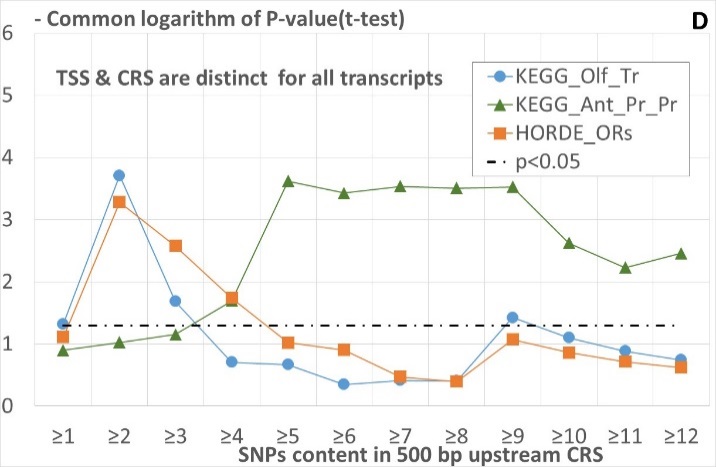 |
| 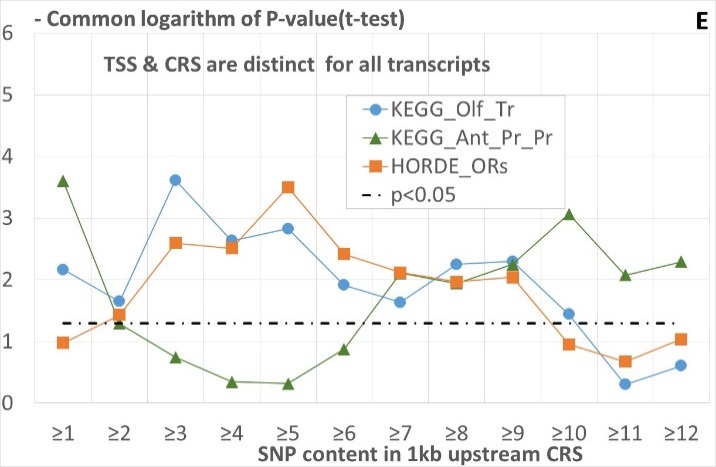 | Figure S2*. The significances of the *t* tests (Y axis) that compare the SNP contents in upstream regions of transcripts of three groups with that for whole genome dataset as a function of the threshold of SNP content (X axis). The *t-* test was applied as described in *Materials and Methods.* Panels A and B present the analyses for 1-kb and 500-bp long regions upstream annotated TSSs, respectively. Panels C, D and E present the same analysis for 500-bp, 500-bp and 1-kb long regions respective to annotated CRSs. Analyses shown in panels B, D, and E were done only for transcripts with annotation of 5’-UTRs. Table 1 shows the sizes and specifications for all datasets of transcripts. |

* In the *Results* section the cases that are presented on panels from A to E were denoted as 5’UTR≥0_(-1kb; TSS), 5’UTR>0_(-500; TSS), 5’UTR≥0_(-500; CRS), 5’UTR>0_(-500; CRS) and 5’UTR>0_(-1kb; CRS), respectively.

**Part 3. Some details of SNP calling performed by 1000 Genomes Project Consortium.**

In both phase 1 and pilot stages of the 1000 Genomes project the special filter *depth threshold* was applied to remove miscalling of SNPs based on the mapping of paralogous sequences (1000 Genomes Project Consortium et al., 2010). The authors rejected sites that were more than twice or less than half the mean depth of coverage across samples in the trio and low coverage projects. In the exon project, the depth of coverage was too variable and this filter was not applied. The filters were described in the section *SNP Calling* of Supplementary Information to the 1000 Genomes Project Consortium report. For example, as it was described in the section *Assessment of the accessible genome in Phase 1* of Supplementary Information to the 1000 Genomes Project report, the average total depth of coverage across Phase I samples was 5132. Thus, sites with a depth of coverage of <2566 or >10264 were rejected (1000 Genomes Project Consortium et al., 2012). As far as we used in our analysis only promoter sequences, the lack of depth threshold filter in case of exon data should not influence our results.

The filters on coverage and fraction of reads with low mapping quality described above lead to the exclusion of a substantial fraction of sites in the genome. The SNP calling procedure removed from analysis genomic regions with many ambiguously placed reads or unexpectedly high or low numbers of aligned reads (1000 Genomes Project Consortium et al., 2010). For both the pilot project and phase 1 analysis the 1000 genomes project created what they defined as accessibility masks. The pilot mask showed that only 85% of the genome is accessible to accurate analysis with the short read technology. The remaining 15% is either repeats or segmental duplications. For phase 1 analysis, using the pilot callability criteria, 94% of the genome was accessible (http://www.1000genomes.org/faq/why-only-85-genome-assayable). We are sure that if the upstream regions of olfactory receptor genes had any assembly problems their SNPs would certainly be eliminated from the final SNP set.

**References**

1. 1000 Genomes Project Consortium, Abecasis, G.R., Altshuler, D., Auton, A., Brooks, L.D., Durbin, R.M., Gibbs, R.A., Hurles, M.E., McVean, G.A. (2010) A map of human genome variation from population-scale sequencing. *Nature.* 467, 7319, 1061-73. doi: 10.1038/nature09534.
2. 1000 Genomes Project Consortium, Abecasis, G.R., Auton, A., Brooks, L.D., DePristo, M.A., Durbin, R.M., Handsaker, R.E., Kang, H.M., Marth, G.T., McVean, G.A. (2012) An integrated map of genetic variation from 1,092 human genomes. *Nature.* 491, 7422, 56-65. doi: 10.1038/nature11632.
